# The effect of psychosocial interventions on depression, anxiety, and quality of life in hemodialysis patients: a systematic review and a meta-analysis

**DOI:** 10.1007/s11255-022-03374-3

**Published:** 2022-10-01

**Authors:** Serena Barello, Gloria Anderson, Marta Acampora, Caterina Bosio, Elena Guida, Vincenzo Irace, Carlo Maria Guastoni, Barbara Bertani, Guendalina Graffigna

**Affiliations:** 1 EngageMinds HUB–Consumer, Food and Health Engagement Research Center, Università Cattolica del Sacro Cuore, Milan, Italy; 2 Department of Psychology, Università Cattolica del Sacro Cuore, L.go Gemelli 1, 20123 Milan, Italy; 3 Department of Biomedicine and Prevention, University of Rome Tor Vergata, Rome, Italy; 4 Associazione Nazionale Emodializzati Emodialisi e Trapianto–ONLUS, Rome, Italy; 5 UO Nefrologia ASST Ovest Milanese, Legnano, Italy; 6 Ordine degli Psicologi della Lombardia, Milan, Italy; 7 Faculty of Agriculture, Food and Environmental Sciences, Università Cattolica del Sacro Cuore, via Milano 24, 26100 Cremona, Italy

**Keywords:** Hemodialysis, End-stage renal diseases, Depression, Anxiety, Psychosocial intervention, Quality of life

## Abstract

**Purpose:**

Hemodialysis has become a standard therapy for adults with end-stage renal diseases. Adults undergoing hemodialysis have to cope with unique psychological issues that make their care journey particularly fatiguing. In this systematic review and meta-analysis, we aimed to summarize and evaluate the effects of psychosocial interventions on the reduction of anxiety and depression in adults with HDs.

**Methods:**

We included randomized controlled trials and quasi-experimental studies that measure change in depression, anxiety, and quality of life.

**Results:**

We identify three categories of psychosocial interventions delivered to adults undergoing hemodialysis. Based on our analysis, there was a medium effect of psychosocial intervention on depression (SMD − 0.85, 95%CI − 1.17; − 0.52, *I*^2^ = 80%, *p < *0.01) and anxiety (SMD − 0.99, 95%CI − 1.65; − 0.33, *I*^2^ = 88%, *p < *0.01) in adults undergoing hemodialysis.

**Conclusions:**

Psychosocial interventions, such as psychological support or relaxation-based therapy, seems all to reduce depression and anxiety in adults undergoing HD. Preliminary evidence suggests that there may be a benefit of psychosocial interventions on the quality of life for adults undergoing HD.

**Supplementary Information:**

The online version contains supplementary material available at 10.1007/s11255-022-03374-3.

## Introduction

End-stage renal disease (ESRD) leads to renal replacement therapy, which includes hemodialysis (HD) treatment. Although HD has become a standard therapy for ESRD, people with HD have to cope with unique psychological issues that make their care journey particularly fatiguing [1–3]. Indeed, HD can make individuals experience different emotions, such as stress, anxiety and depression as well as hopes and gratitude [1, 4]. According to Goyal et al. [5], 45% of adults undergoing HD have some degree of psychological issues [5]. The prevalence of depression and anxiety symptoms in the dialysis population is high and is associated with increased mortality [6–8]. Moreover, many evidences indicates that people with ESRD treated with hemodialysis have impaired health-related quality of life (HRQoL) when compared to the general population [9–11].

In many clinical settings, interventions for reducing psychosocial distress in adults undergoing HD are not standard care [12]. This suggests that current health care policies often fail to sufficiently embrace the needs of vulnerable people with ESRD undergoing HD. Indeed, as a complementary therapy to pharmacological strategies, psychosocial interventions are recommended to improve both clinical and psychosocial outcomes of kidney disease care [13]. However, the efficacy of psychosocial interventions is still debated by some scholars. Therefore, whether psychosocial interventions effectively promote the psychological well-being and quality of life in adults undergoing HD remains to be confirmed. This issue has not been summarized in any previous systematic review or meta-analysis.

The present systematic review and meta-analysis aim to summarize and evaluate the effects of psychosocial interventions on the reduction of anxiety and depression in adults with HD. A secondary aim is to describe and assess the impact of such interventions on adults undergoing HD's quality of life. The results can provide medical facilities and dialysis centers with an evidence-based basis for establishing psychosocial interventions in kidney care settings.

### Objectives

The objectives of the present review are:

1. To identify studies reporting psychosocial interventions aimed at reducing depression and anxiety in adults undergoing HD and at synthesizing their key characteristics.
2. To assess the efficacy of psychosocial interventions at reducing depression and anxiety or enhancing the quality of life in people undergoing HD.
3. To identify whether differences in efficacy exist depending on the type of administered intervention, the deliverer, or the intervention's length.

## Methods

Using a priori published protocol on PROSPERO (CRD42020219072), we conducted a systematic review following the Cochrane Handbook for Systematic Reviews of Interventions and reported the findings according to the Preferred Reporting Items for Systematic review and Meta-Analyses (PRISMA) criteria [14, 15].

### Eligibility criteria

We considered eligible: (i) all the randomized controlled trials (RCTs) or quasi-randomized controlled trials (quasi-experimental) conducted in any healthcare infrastructures; (ii) all the psychosocial interventions administered on adults, defined as any intervention used to modify behavior, emotional state or feeling. We defined psychosocial interventions according to criteria used in previous Cochrane reviews of such interventions for long-term diseases [13, 16, 17]. This definition included any intervention involving systematic treatment with some form of feedback or review, including psychotherapy, psychoeducation, and interventions containing elements, such as education, goal-setting, cognitive restructuring, behavioral strategies, relaxation training, stress management, or support groups. Multimodal interventions, consisting of both psychological and other therapeutic procedures (e.g., exercise), were also included, if one or more social–psychological therapeutic technique was clearly evident. Both individual and group-focused interventions were included, regardless of delivery mode; (iii) interventions involving people affected from CKD of any etiology and duration, stages 3–5 (eGFR falling below 60 ml/min/1.73m^2^), undergoing hemodialysis, at home or an ambulatory; (iii) studies that stated a validated self-reported measure of depression and/or anxiety; (iv) studies that compared an intervention group with usual care or placebo. Only studies available in the full text were included to ensure sufficient appraisal and review.

### Search strategy

Studies were identified by searching the following databases: PsycInfo, Medline, Embase, Web of Science (WoS), Cumulative Index to Nursing and Allied Health Literature (CINAHL), and Cochrane Central Register of Controlled Trials (CENTRAL) and Scopus. In addition, the eligible articles’ references were screened to identify any relevant articles missed by the electronic and manual searches. We screened all the available articles in the databases until the 30th of June 2022.

The search strategy included a combination of MeSH terms and keywords with appropriate Boolean operators (Supplemental File 1). The quality of the search strategy, tailored for each database, was developed with the aid of a medical librarian with expertise in database searches for reviews. No time limits were applied across the searches, and all the articles were screened with the support of the reference's manager Zotero (v5.0). The search was restricted to articles published in English, Italian or Chinese.

### Study selection

Four authors (SB, MA, GA, EG) independently reviewed the articles by title, abstract and full text to identify all the eligible studies. Disagreements were solved by involving one or more additional authors (SB, MA, GA, EG). We included all the RCTs and quasi-experimental on adults with CKD that assessed the efficacy of a psychosocial intervention on anxiety or depression. We excluded: (i) all the uncontrolled intervention studies, or case series studies, or observational studies; (ii) all the studies on children or adolescents; (iii) all the studies on people who were enrolled for kidney transplantation or had just received it; (iv) primary stages of CKD when eGFR is still normal or slightly reduced (eGFR above 60 ml/min/1.73 m^2^); (v) all the pharmaceutical interventions, the exclusive exercise interventions, the spiritual interventions, alternative medicine interventions and interventions revolving around making a specific change in the diet; (vi) all the studies that reported a dichotomous measurement of anxiety or depression.

### Data collection and extraction process

Four authors (SB, MA, GA, EG) independently extracted the data for the qualitative synthesis, the quantitative synthesis (SB, MA, GA, EG) and the Risk of Bias assessment (SB, MA, GA, EG). Data from the included studies were extracted using a pre-designed data extraction form developed from the data collection checklist by the Cochrane Effective Practice and Organisation of Care Review Group (EPOC). Key aspects of data extraction included: author, publication date, country, demographic or disease characteristics, study design, intervention description, the deliverer of the intervention, data analyses, and results for the outcomes of interest. Where missing data were encountered, corresponding authors were contacted to retrieve further information. We found no papers reporting on the same trial, so only unique evidence was gathered.

### Outcome data

Validated psychometric self-report measures of depression and anxiety were extracted, such as the Beck Depression Inventory (BDI) scale or the Hospital Anxiety and Depression Scale (HADS). Whether there were missing or unclear data, we tried to contact the authors for further clarifications. To synthetize the key characteristic of the psychosocial interventions, two authors independently extracted the tools and strategies applied in each intervention. At the end of the synthesis process, all the authors reviewed and agreed on the developed categorization.

### Quality and risk of bias assessments

#### Within-study bias

Risk of bias (RoB) for the RCTs was assessed by two authors using the Revised Cochrane Risk of Bias (RoB2) assessment tool [18]. RoB2 assesses the RoB in RCTs through five domains: bias arising from the randomization process, bias due to deviations from the intended interventions, bias due to missing outcome data, bias in the measurement of the outcome and bias in the selection of reported results. Each study was classified into ‘low’, ‘high’, or ‘some concerns’ RoB for each domain. Disagreements were solved by discussion and consensus, and in cases, where consensus could not be reached, the opinion of a third reviewer was sought.

RoB for the non-randomized studies of interventions was assessed by two authors using the Cochrane Risk of Bias in Non-Randomized Studies of Interventions (ROBINS-I) tool [19]. ROBINS-I assesses the RoB in quasi-experimental through seven domains: bias due to confounding; bias in the selection of participants into the study; bias in classification of interventions; bias due to deviations from intended interventions; bias due to missing data; bias in the measurement of outcomes; bias in the selection of reported results. Each study was classified into “low”, “moderate”, “serious,” or “critical” Rob for each category.

Where information reported in the paper was insufficient or unclear, authors were contacted. RoB ratings for both RCTs and quasi-experimental were presented using Robvis (Cochrane Collaboration; Supplemental Files 2).

### Between-study bias

The risk of publication bias was evaluated to control whether studies reporting statistically significant results were more likely to be published, potentially overestimating the real effect size [20]. To assess the presence of publication bias, a funnel plot for symmetry was constructed, with asymmetry suggesting the possibility of publication bias. Given the subjective nature of interpreting funnel plots, Egger's test to measure funnel plot asymmetry was used as an objective test to support the conclusions [21].

### Data analysis and synthesis

A narrative synthesis of the included studies was conducted to provide a conceptual summary of the findings across all the included studies. A quantitative synthesis was conducted using R’s metan package (v.1.11.0). We summarized the findings using descriptive statistics, including mean, median, standard deviations (SDs) and frequencies (percents). A DerSomonian–Laird (DerSimonian & Laird) random-effects meta-analysis with 95% confidence intervals was performed. A random-effects model output was selected as moderate or high clinical and methodological heterogeneity was expected. Forest plots were provided for anxiety, depression and quality of life. Since the variables of interest were all continuous but reported using different weight units, a standardized mean difference (SMD) was computed.^14^ When standard deviation were not reported, we converted standard errors (SEs), 95% confidence intervals (Cis) and interquartile ranges (IQRs) to SDs using the formulas suggested in the Cochrane Handbook for Systematic Reviews and Meta-Analysis. The Hedges’G (*g*) form of the SMD was used. For anxiety and depression, negative SMDs indicate a difference in results favoring the intervention. A SMD of 0.20 is considered a small, 0.50 medium and > 0.80 a large effect [22]. Depression and anxiety were recoded where necessary, so low scores indicate lesser depression or anxiety. Cochrane's *Q* chi-squared test and *I*^2^ statistic were used to assess the level of statistical heterogeneity. A *p* value of < 0.1 and a *I*^2^ > 75% were considered significant. Sensitivity analysis were performed after the detection and the examination of outliers, using R’s *InfluenceAnalysis* function [23]. Subgroup analyses were also conducted to assess if effect sizes vary according to the following characteristics: (i) type of intervention, (ii) deliverer, (iii) length of the intervention (more or less than 5 weeks), (iv) type of sessions (group vs. individual). Sensitivity and subgroup analyses were conducted on the most complete data set, using endpoint-point data of each study. If there were fewer than three studies for each subgroup, we decided not to pool the subgroup [24].

### Certainty assessment

The overall certainty of evidence across studies was rated using the Grading of Recommendations Assessment, Development and Evaluation (GRADE) working group guidelines. According to the corresponding evaluation criteria, the quality of evidence was classified into four categories: high, moderate, low and very low.

## Results

### Results of the search

**Fig. 1:**
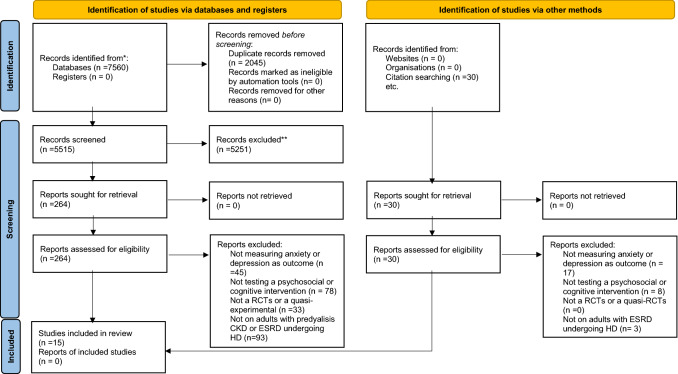
Search strategy

The electronic searches resulted in 7320 citations. After the merging of the duplicates, 5324 citations were screened. Four authors (SB; GA; EG; CB) screened the titles and abstracts and identified 193 records as potentially relevant, which were retrieved for further assessment. After full-text screening, 15 studies were deemed eligible according to the inclusion and exclusion criteria shown in Fig. 1. The most common reasons for exclusion after full-text appraisal were: (i) not measuring the incidence of depression or anxiety as an outcome [25–31], (ii) not testing a psychosocial or cognitive intervention [32–35], and (iii) not a RCTs or quasi-experimental controlled study design [36–38]. Out of the 15 trials included in our systematic review and meta-analysis, 10 were RCTs, and five were quasi-experimental. All the RCTs randomized the participants at individual levels. A summary of the study characteristics and demographics is presented in Table 1 [39–54].

**Table 1:** Characteristics of the included studies

Trial	Years	Country	Design	*N*	Participants	Assessment	Follow-up	Outcomes	Scale
Bahamani et al. [39]	2015	Iran	Quasi-experimental	22	Women Patients with CKD under hemodialysis treatment	Baseline 3 months	None	Depression ↑	BDI-II-21
Beizaee et al. [40]	2018	Iran	Quasi-experimental	80	Patients with CKD under hemodialysis treatment Mean age 47.2 years Most of the sample had low educational attainment Predominantly male (33 female; 47 male)	Baseline 1 month	None	Depression ↑ Anxiety ↑	HADSS
Duarte et al. [41]	2009	Brazil	RCT	85	Patients with CKD under hemodialysis treatment Mean age 53.2 years Most of the sample had low educational attainment Predominantly female (50 female; 35 male)	Baseline 3 months	Yes, at 9 months	Depression ↑ Quality of life ↑	BDI KDQOL-SF
Espahbodi et al. [42]	2013	Iran	RCT	55	Patients with CKD under hemodialysis treatment Mean age 50.7 years Predominantly female (28 female; 27 male)	Baseline 1 month	None	Depression ↑ Anxiety	HADSS
Heshmatifar et al. [44]	2015	Iran	RCT	70	Patients with CKD under hemodialysis treatment Mean aged 62.3 years Most of the sample had low educational attainment Predominantly male (35 male; 35 female)	Baseline 1 month	None	Depression ↑	BDI
Irajpour et al. [45]	2019	Iran	Quasi-experimental	65	Patients with ESRD receiving hemodialysis treatment Most of the sample had low educational attainment (Gender and age distribution not reported)	Baseline 2 months	None	Depression ↑ Anxiety ↑	DASS-42
Kim [46]	2018	South Korea	Quasi-experimental	48	Patients with CKD under hemodialysis treatment Mean age 58.7 years Most of the sample had high educational attainment Predominantly male (29 male; 19 female)	Baseline 2 months	None	Depression	BDI
Lerma et al. [47]	2018	Mexico	RCT	49	Patients with ESRD receiving hemodialysis treatment Mean age 41.75 years Most of the sample had high educational attainment Predominantly female (26 female; 23 male)	Baseline 1 month	Yes, at 2 months	Depression ↑ Anxiety Quality of life ↑	BDI BAI QoL Profile in the Chronically Ill
Lii et al. [48]	2007	Taiwan	RCT	48	Patients with ESRD receiving hemodialysis treatment Most of the sample had high educational attainment Predominantly female (35 female; 33 male)	Baseline 1 month	None	Depression ↓ Health related quality of life ↑	BDI MOS SF-36
Mehrabi et al. [49]	2017	Iran	RCT	50	Patients with ESRD receiving hemodialysis treatment (Gender and age distribution not reported)	Baseline 1 month	None	Depression ↓ Anxiety ↓	DASS-21
Rahimpour et al. [50]	2016	Iran	Quasi-experimental	50	Patients with CKD under hemodialysis treatment Mean age 47.82 years Predominantly male (24 female; 26 male)	Baseline 1 month	None	Depression ↓ Anxiety ↓	DASS-21
Tsai et al. [51]	2015	Taiwan	RCT	64	Patients with CKD receiving hemodialysis treatment Mean age 63.1 years Most of the sample had low educational attainment Predominantly female (number of female not reported)	Baseline 1 month	None	Depression ↓ Health related Quality of life ↑	BDI-II MOS SF-36
Tsay et al. [53]	2003	Taiwan	RCT	50	Patients with ESRD receiving hemodialysis treatment Mean age 51.18 years Most of the sample had low educational attainment Predominantly female (20 male; 30 female)	Baseline 1 month	None	Depression	BDI
Tsay et al. [52]	2005	Taiwan	RCT	57	Patients with ESRD receiving hemodialysis treatment Mean age 50.72 years Most of the sample had high educational attainment Predominantly female (30 female; 27 male)	Baseline 1 month 3 months	None	Depression ↓ Quality of life ↑	BDI MOS SF-36
Zhianfar et al. [54]	2020	Iran	RCT	70	Patients with ESRD receiving outpatient hemodialysis treatment Middle aged (40–60 years) 77.27% had low educational attainment Predominantly female (38 female; 32 male)	baseline 1 month 3 months	None	Depression Quality of life ↑	BDI-SF WHOQOL-SF

↓ = a statistically significant decrease of the outcome; ↑ = statistically significant increase of the outcome

### Characteristics of the included studies

Fifteen trials were included, with a total of 837 participants [39–54]. Trial sample sizes ranged from 22 to 94 participants. The trials were conducted in six different countries (Table 1), with the Middle East (e.g., Iran) and Asian (e.g., Taiwan) countries being the most prevalent. The trials were published between 2003 and 2020, with 60% of the included studies published in the last 5 years. With regard to demographic characteristics of the samples, females (58%) were slightly more prevalent than males across the studies. The samples’ mean age ranged from 41.75 years [47] to 63.01 years [51], with a weighted mean age of 51.5 years. Four studies did not report the mean age for the participants, and three studies provided little information on the sample characteristics (Table 1). All the included studies involved dialysis patients, with a mean length of dialysis ranging from 3 months to more than 5 years. Few studies reported the main cause of the disease, [41, 46, 51] with diabetes and high blood pressure being the most common. Both depression and anxiety were a primary outcome in all the included studies. Most of the studies used or the Beck Inventory Scale (BDI) or Hospital Anxiety and Depression scale (HADS) to measure depression, while only three studies used the Depression Anxiety Stress Scales (DASS). Instead, anxiety was prevalently measured with either HADS or DASS. Only four studies assessed outcomes on multiple timing after the intervention, while the remaining studies used a single assessment (Table 1). Twelve studies assessed outcomes shortly after the intervention (less than 6 weeks), while the other assessed outcomes at longer intervals following the intervention for a maximum period of 3 months (Table 1). Only two studies stated a follow-up, ranging from 2 up to 9 months [41, 47]. Out of the 15 studies, 13 found significantly lower depression scores in the intervention groups compared to the control groups.

### Interventions

Great variability existed in relation to intervention contents and structure, as shown in Table 2. Frequency and duration of treatment sessions varied considerably among the trials, with a minimum number of four sessions up to 12 sessions (Table 2). The length of the session varied from four to 12 weeks (Table 2). All the interventions were delivered face-to-face, either individually and/or in a group format (Table 2). Across the interventions, the sessions lasted between 15 and 120 min. The interventions were usually delivered immediately before or the day after a dialysis treatment. The majority of the sessions were held in dialysis centers or at the participant's home (Table 2). Many studies used tools (e.g., booklets) or devices (e.g., laptops) to enhance the interventions' effect. The most common provider for the interventions were nurses, followed by multidisciplinary teams, and finally psychologists (Table 2). Only one study [45] used a peer (i.e., an expert patient) as the main provider for the intervention. When the interventions were coded (Table 2), the following categories were defined: psychoeducation, psychological support and relaxation-based therapy. The psychoeducational intervention aimed to increase the participant's health literacy and develop goal-setting strategies and/or problem-solving skills (Table 2). The interventions coded as psychological support interventions were generally aimed to promote the patients' acceptance and elaboration of the disease; they were the majority and mainly cognitive–behavioral interventions (CBTs), with only one study testing a Schneider's hope therapy intervention [50]. The relaxation-based interventions aimed to increase self-regulation and emotional, cognitive, and behavioral flexibility were the most heterogeneous category, enclosing different techniques, such as Benson relaxation-based methods or guided imagery intervention or breathing training (Table 2).

**Table 2:** Summary of the interventions

Study	Intervention	Comparator	Approach	Length	Provider	Type of session	Setting
Bahamani et al. [39]	Psychological support	Usual care	CBT	12 sessions of 90 min twice per week	Psychologist	ns	ns
Beizaee et al. [40]	Relaxation-based therapy	Usual care	Guided imagery	Three sessions for 4 weeks	Psychologist	Face-to-face	Dialysis center
Duarte et al. [41]	Psychological support	Usual care	CBT	12 weekly sessions of 1.3 h for 3 months	Psychologist	Face-to-face Group sessions	Dialysis center
Espahbodi et al. [42]	Psychoeducation	Usual care	Self-management education	One hour every day before the dialysis appointment	Multidisciplinary Team	Face-to-face Group sessions	
Heshmatifar et al. [44]	Relaxation-based therapy	Usual care	Benson’s relaxation method	Twice a day for 20 min over 1 month	Multidisciplinary Team	Face-to-face Individual sessions	Dialysis center; patient's home
Irajpour et al. [45]	Psychoeducation	Usual care	Self-management education	Eight session of 120 min for 8 weeks	Peer	Face-to-face Individual sessions	Patient's home
Kim [46]	Psychoeducation	Usual care	Self-management education	Eight sessions of 50 min for 8 weeks	Nurse	Face-to-face Individual sessions	ns
Lerma et al. [47]	Psychological support	Usual care	CBT	Five sessions of two hours for 5 weeks	Psychologist	Face-to-face Group sessions	ns
Lii et al. [48]	Psychological support	Usual care	CBT	Eight sessions of 2 h for 8 weeks	Nurse	Face-to-face Group sessions	Dialysis center
Mehrabi et al. [49]	Psychological support	Usual care	Fordyce’s happiness training	Six sessions of 20 min for 8 weeks	Nurse	ns	ns
Rahimpour et al. [50]	Psychological support	Group discussion	Schneider's hope therapy program	Eight sessions of 1–1.5 h for 8 weeks	Nurse	Face-to-face Individual sessions	ns
Tsai et al. [51]	Relaxation-based therapy	Waiting list	breathing training and relaxation	Eight sessions for 4 weeks	Nurse	Face-to-face Individual and group sessions	Dialysis center
Tsay et al. [53]	Psychoeducation	Information package	Self-management education	12 sessions for 4 weeks	Nurse	Face-to-face Individual sessions	Dialysis center
Tsay et al. [52]	Psychological support	Usual care	CBT	ns	Multidisciplinary Team	Face-to-face Group sessions	Dialysis center
Zhianfar et al. [54]	Psychological support	Usual care	CBT	Eight sessions of 90 min	Multidisciplinary Team	Face-to-face + telephone-based support Group sessions	Dialysis center

*ns* not stated; *CBT* cognitive behavioral therapy

In relation to the comparison group, most studies compared the intervention to usual care or handbook provision (Table 2), with only one study using an enhanced comparison group [50].

### Meta-analysis

#### Primary outcomes

##### Depression

**Fig. 2:**
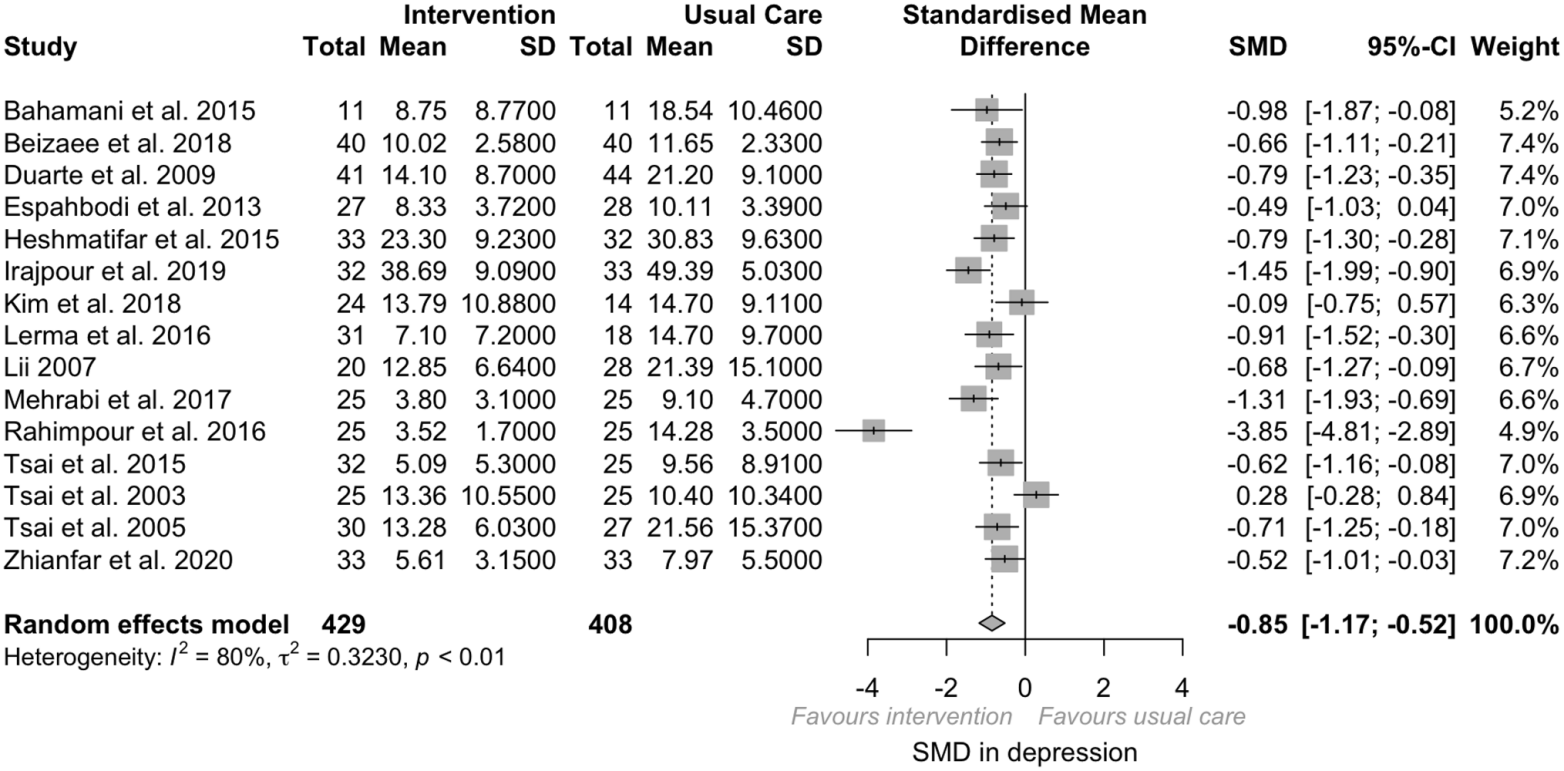
Depression forest plot

According to our analysis, relying on endpoint data of each trial, there was a large significant effect size of psychosocial interventions on depression across the 15 RCTs and quasi-experimental included (*N* = 837; SMD = − 0.85, *p < *0.001; 95% CI − 1.17; − 0.52, *I*^2^ = 79.7%, *p < *0.001). Psychosocial interventions were associated with a significant reduction in depression scores (Fig. 2). Significant heterogeneity existed between estimates (*I*^2^ = 79.7%, *p < *0.001). However, once we removed the studies at high or serious RoB, a medium significant effect size was retained (SMD = − 0.67; *p < *0.001; 95%CI = − 0.97; − 0.371), and the heterogeneity was reduced (*I*^2^ = 66.9%, *p < 0.0*01). When the RCTs and the quasi-experimental were analyzed separately, the effect ranged from medium in RCTs (SMD = − 0.64; *p < *0.001; 95%CI − 0.88; − 0.41, *I*^2^ = 47.5%, *p* = 0.04) to large in quasi-experimental (SMD = − 1.35; *p* = 0.007, 95% CI − 2.34; − 0.36, *I*^2^ = 91.2%, *p < *0.001). However, high heterogeneity existed in quasi-experimental (*I*^2^ = 91%, *p < *0.001), probably due to the existence of confounding, while RCTs had low heterogeneity (*I*^2^ = 47.5%, *p* = 0.04).

Looking at the endpoint data separately, at 1 month a large significant effect size of psychological interventions on depression was pooled (*k* = 8, SMD = − 0.94; *p < *0.001; 95%CI = − 1.52; − 0.37, *I*^2^ = 88%, *p* = 0.001). A medium significant effect size of psychological interventions on depression was retained at two (*k* = 3, SMD = − 0.83; *p < *0.001; 95%CI = − 1.60; − 0.07, *I*^2^ = 79%, *p* = 0.001) and three (*k* = 4, SMD = − 0.71; *p < *0.001; 95%CI = − 0.98; − 0.44, *I*^*2*^ = 0%, *p* = 0.79) months.

##### Anxiety

**Fig. 3:**
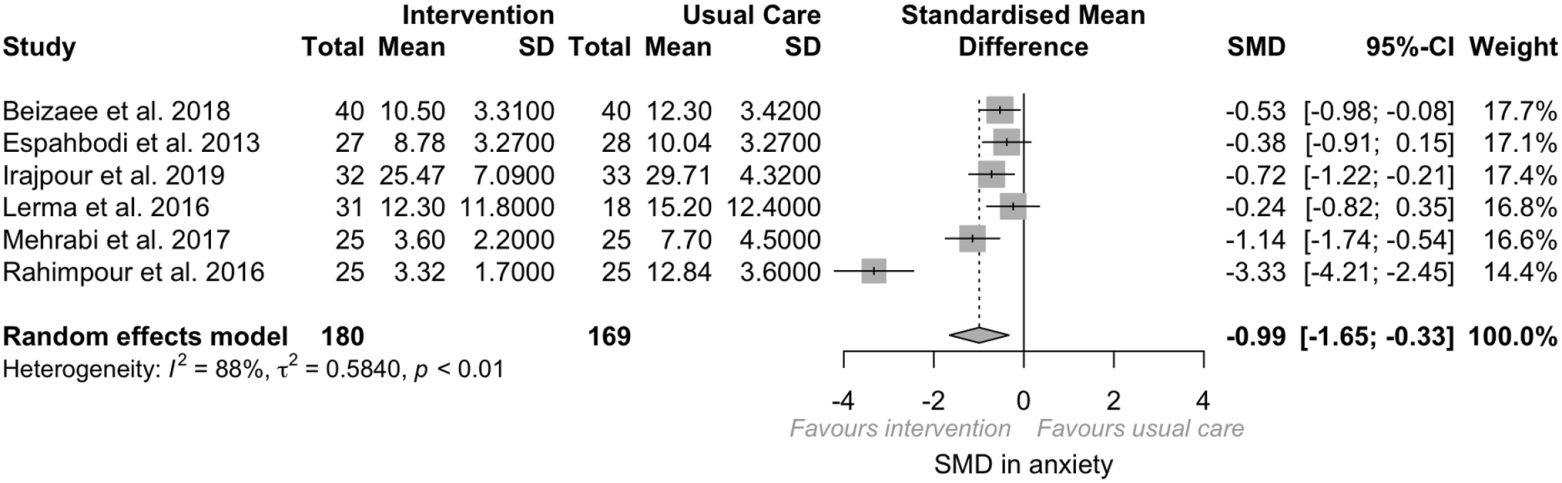
Anxiety forest plot

There was a large significant effect size of psychosocial interventions on anxiety across the six RCTs and quasi-experimental included (*N* = 349; SMD = − 0.99, *p* = 0.003; 95% CI − 1.64; − 0.33, *I*^2^ = 87.7%, *p < *0.001). Psychosocial interventions were associated with a significant reduction in anxiety scores (Fig. 3). The effect was retained with low heterogeneity (SMD = − 0.64, *p < *0.001; 95% CI − 0.97; − 0.30, *I*^2^ = 37.6%, *p* = 0.18) after the remotion of studies at high or serious RoB. When the RCTs and the quasi-experimental were analyzed separately, as for the pooled estimates of depression, medium heterogeneity was observed in the RCT (SMD = − 0.57, *p* = 0.03, 95% CI − 1.1, − 0.04, *I*^2^ = 61.4%, *p* = 0.07) and high heterogeneity in the quasi-experimental (SMD = − 1.46, *p* = 0.03; 95% CI − 2.80; − 0.12, *I*^2^ = 93.8%, *p < *0.001).

#### Secondary outcomes

##### Quality of life

Only three studies reported the effect of psychosocial intervention on the quality of life of participants undergoing dialysis. Overall, compared to the control group, a significant improvement in quality of life was observed (*N* = 189, SMD = 0.65, *p* = 0.001; 95% CI 0.35; 0.95) with no evidence of heterogeneity (*I*^2^ = 0, *p* = 0.85).

##### Subgroup analyses

Exploratory subgroup analyses were conducted to identify potential moderators of the pooled effect size of social–psychological interventions on depression and anxiety. Some variables used in these analyses were selected a priori (e.g., type of intervention); others, such as provider and length of the intervention, were selected post-hoc. None of these moderator effects were statistically significant, even if quantitative interaction existed in all the subgroups [14]. All the subgroup analyses are reported in Table 3.

**Table 3:** Subgroup analyses

Subgroup	SMD (95% CI)	Number of studies (*k*)	*P* value for interaction
Depression			
Type of intervention			0.19
Psychological support intervention	− 1.12 (− 1.81; − 0.62)	7	
Psychoeducational intervention	− 0.49 (− 1.07; 0.09)	5	
Relaxation-based intervention	− 0.68 (− 0.97; − 0.40)	3	
Deliverer			0.32
Nurses	− 0.99 (− 1.88; − 0.1)	6	
Psychologists	− 0.78 (− 1.05; − 0.51)	4	
Multi-disciplinary team	− 0.62 (− 0.88: − 0.37)	4	
Lenght of the intervention			0.42
Longer than 5 weeks	− 1.15 (− 1.89; − 0.42)	6	
Less than 5 weeks	− 0.68 (− 1.05; − 0.32)	7	0.5
Type of sessions			
Individual sessions	− 1.13 (− 2.21; − 0.04)	5	
Group sessions	− 0.67 (− 0.89; − 0.46)	6	

##### Sensitivity analyses

**Fig. 4:**
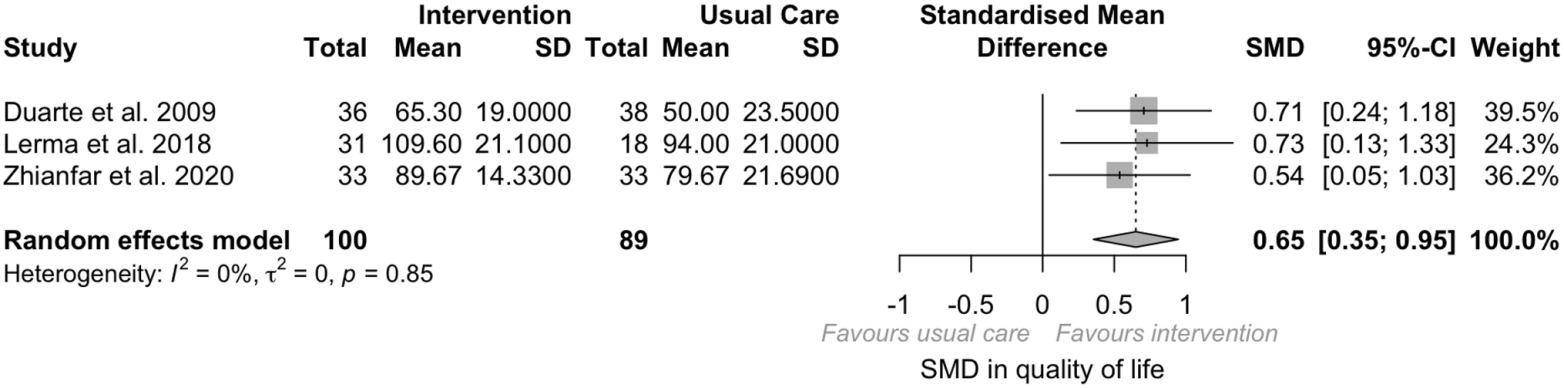
Quality-of-life forest plot

Sensitivity analyses were conducted on the results of the influence analysis. Indeed, two outliers were identified and removed (Supplemental File 3) [50, 51]. Once removed, a medium significant effect size of psychological intervention on depression (SMD = − 0.76; 95% CI − 0.94; − 0.58; *I*^*2*^ = 25%, *p* = 0.19) and anxiety (SMD = − 0.59; 95% CI − 0.87; − 0.31; *I*^2^ = 28%, *p* = 0.24), both with low heterogeneity, was observed (Fig. 4).

#### RoB of the included studies

Fifteen studies were assessed for the RoB, 10 with the RoB2 and five with the ROBIN-I. As can be seen from Supplemental File 2, the studies were predominantly of poor quality, with a strong prevalence of serious or high-risk judgements. The poor description of the intervention [39, 49, 51] or of the sample characteristics at baseline [39, 43, 47–50], as well as the risk of selective reporting, significantly lowered the methodological quality of the majority of the included studies (Table 4).

**Table 4:** Certainty of evidence

Quality assessment						Summary of findings			
Outcomes	Risk of Bias	Inconsistency	Indirectness	Imprecision	Publication bias	Number of intervention/control	SMD (95%CI)	Heterogeneity (*I*^2^)	Quality of evidence
Depression	No serious limitations	Very serious limitations	No serious limitations	No serious limitations	No serious limitations	429/408	− 0.85 (− 1.17;− 0.52)	80%	Low
Anxiety	No serious limitations	Very serious limitations	No serious limitations	No serious limitations	No serious limitations	180/168	− 0.99 (− 1.65; − 0.33)	88%	Low
Quality of life	No serious limitations	No serious limitations	No serious limitations	Serious limitations	No serious limitations	100/89	0.65 (0.35; 9.95)	0%	Moderate

For the RCTs, the main reasons for high or some concerns RoB were the domains of bias arising from the randomization process or in the selection of reported results (Supplemental File 2). Many studies provided few details on the randomization process, such as the type of randomization (e.g., simple, block or stratified) or on the sample characteristics at baseline. For the quasi-experimental, the main reasons for moderate or serious RoB were biased due to confounding in the classification of interventions or the measurement of outcomes (Supplemental File 2).

#### RoB across studies

The somewhat asymmetrical funnel plot, with few studies in the bottom left of the plot, suggested that the review may be subjected to publication bias. However, a non-significant Egger's statistical test (bias = − 5.66; 95% CI − 11.13 to − 0.19; *p* = 0.063) was computed, indicating no substantial asymmetry in the funnel plot.

### Grading of evidence

To assess the quality of evidence for outcomes, the Grade framework was performed and determined the effect of psychological intervention to be of moderate quality on quality of life (Table 3). The evidence about depression and anxiety was downgraded to low (Table 3).

## Discussion

In our systematic review and meta-analysis, we analyzed a total of 15 studies involving 837 adults undergoing HD. We found three types of psychosocial interventions that involved adults undergoing HD: psychological support, relaxation-based therapy, and psychoeducational intervention. A positive effect of psychosocial interventions on depression, anxiety, and quality of life of adults undergoing HD was observed. The decrease in both depression and anxiety scores was kept after excluding studies at high risk of bias. Our findings are consistent for both the RCTs and the quasi-experimental and are coherent with other meta-analyses [55, 56]; however, our meta-analysis is the first study to involve such many studies and different psychosocial intervention types for adults undergoing HD.

Psychological support interventions, such as CBT or relaxation-based therapy, were the most commonly developed psychosocial interventions to reduce depression and anxiety in people with HD. Multiple factors affect the quality of life of adults undergoing HD, such as impairment of body functions and structures, limitations in activity, restrictions in life participation and sexual disorders [57, 58]. The use of avoidance coping mechanisms to deal with all these issues is associated with a worsening of the psychological state of health [59]. Enhancing adaptive coping mechanisms through psychological support interventions effectively improves the mental health of adults undergoing HD and should be recommended. Relaxation-based interventions aiming to improve positive coping mechanisms seem to be almost as effective as CBT in reducing depression in adults with HD [60]. However, we found only three RCTs assessing relaxation-based interventions. Further studies are needed to assess the real size of the effect of the relaxation-based intervention on depression and anxiety and test whether relaxation-based interventions are more effective than psychological support interventions.

Focusing on the interventions' characteristics, most of the interventions were short, delivering only weekly sessions for less than 5 weeks. At 1 month, the pooled estimate for the effect of the psychological interventions on depression appears to be larger than at 3 months. Psychological interventions are often complex in nature, necessitating time to impact the process of illness elaboration and the actual patient's ability to effectively cope with the disease’s mental health effects [61]. Continuous support may enhance the positive coping mechanism, helping adults with HD manage their psychological well-being. Designing and testing psychological intervention longer than 5 weeks may help maintain their positive effects longer; however, this hypothesis has to be taken cautiously and needs further scrutiny. We included different types of interventions, from relaxation-based to CBT, that have different timings and are delivered in different settings.

This systematic review and meta-analysis have some limitations. Most of the included studies have short endpoints and low sample size. Only three RCTs were judged at low risk of bias. More studies with more power and good quality designs are needed to confirm our findings. Moreover, we included very few articles from Europe and none from the USA, which may have led to an overestimation or underestimation of the effect size. We were unable to conduct the subgroup analyses for both anxiety and quality of life due to the low number of articles in each subgroup, which could have made the finding meaningless.

## Implication for practice and future research

Our review suggests there is now moderate certainty that psychosocial interventions reduce depressive and anxiety symptoms for patients undergoing HD when compared to usual care, although few enrolled participants and the short measurement endpoints lead to considerable uncertainty and may not provide sufficient evidence to inform clinical practice. Further research is likely to change the estimated effects of different psychosocial interventions in adults undergoing HD with or without psychological symptoms (depression or anxiety) and increase our certainty of the evidence-based on limitations in existing studies and a paucity of evidence for specific clinical questions. Given the high symptom burden experienced by adults undergoing hemodialysis, together with the prioritization of research informing symptom management, new research initiatives for preventing and treating psychological symptoms would address important clinical uncertainties. Depression and anxiety were assessed using different tools; outcomes data were measured in heterogeneous ways and at different timepoints.

The results of this review suggest that psychosocial interventions and, in particular, psychological support are promising interventions for improving psychological well-being in adults undergoing HD that warrant further research. Based on this review, future studies would increase our certainty about whether these interventions improve patient quality of life. Researchers investigating psychosocial treatments should consider well-designed standardized interventions to provide adequate statistical power to detect outcome measures, blinding of outcome assessment for subjective outcomes, and inclusion of all participants in the outcome assessments regardless of whether they complete the intervention as designed. Future psychosocial intervention studies should be designed to evaluate patient‐centered core outcomes relevant for adults undergoing HD, such as HRQoL, impaired mobility, and inability to participate in life and work that are becoming new priorities to aid in clinical decision‐making.

## Conclusions

This meta-analysis suggests that psychosocial interventions are promising interventions for improving psychological well-being in adults undergoing HD. Psychosocial interventions, such as psychological support or relaxation-based therapy, may reduce depression and anxiety in adults undergoing HD. Preliminary evidence suggests that there may be a benefit of psychosocial interventions on the quality of life for adults undergoing HD.

## Supplementary Information

Below is the link to the electronic supplementary material.

Supplementary file1 (DOCX 137534 KB)

## Data Availability

Author may turn data and materials available if this is the editor wish.
