# Supplementary material for: The effect of psychosocial interventions on depression, anxiety, and quality of life in hemodialysis patients: a systematic review and a meta-analysis
Source: Int Urol Nephrol. 2022 Oct 1;55(4):897–912. doi: 10.1007/s11255-022-03374-3 (PMC10030538; doi:10.1007/s11255-022-03374-3)
Supplement: Supplementary file 1 — Supplementary file1 (DOCX 137534 KB) [file 11255_2022_3374_MOESM1_ESM.docx]

**Supplemental File 1**

**Pubmed**

(("Kidney Disease" OR "Kidney Diseases" OR "Renal Insufficiency" OR "Renal Insufficiencies" OR "Renal Replacement Therapy" OR "Renal Replacement Therapies" OR "hemodialysis" OR "haemodialysis" OR "hemofiltration" OR "haemofiltration" OR "hemodiafiltration" OR "haemodiafiltration" OR "chronic kidney failure" OR "chronic renal failure" OR "predialysis" OR "pre-dialysis" OR "chronic renal" OR "chronic kidney" OR "Renal Disease" OR "Renal Diseases") AND ("motivational interviewing" OR "motivational intervention" OR "counseling" OR "behavioral intervention" OR "behavioural intervention" OR "educational intervention" OR "cognitive intervention" OR "psycho-social intervention" OR "psychosocial intervention" OR "psychotherapy" OR "psychological" OR "educational" OR "psycho-educational" OR "psychoeducational")) AND ("randomised controlled trial" OR "controlled clinical trial" OR "randomized" OR "placebo" OR "randomly" OR "trial" OR "groups")

**Medline**

((Kidney Disease OR Kidney Diseases OR Renal Insufficiency OR Renal Insufficiencies OR Renal Replacement Therapy OR Renal Replacement Therapies OR hemodialysis OR haemodialysis OR hemofiltration OR haemofiltration OR hemodiafiltration OR haemodiafiltration OR chronic kidney failure OR chronic renal failure OR predialysis OR pre-dialysis OR chronic renal OR chronic kidney OR Renal Disease OR Renal Diseases) AND (motivational interviewing OR motivational intervention OR counseling OR behavioral intervention OR behavioural intervention OR educational intervention OR cognitive intervention OR psycho-social intervention OR psychosocial intervention OR psychotherapy OR psychological OR educational OR psycho-educational OR psychoeducational)) AND (randomised controlled trial OR controlled clinical trial OR randomized OR placebo OR randomly OR trial OR groups)

**Web of Science**

(("Kidney Disease" OR "Kidney Diseases" OR "Renal Insufficiency" OR "Renal Insufficiencies" OR "Renal Replacement Therapy" OR "Renal Replacement Therapies" OR hemodialysis OR haemodialysis OR hemofiltration OR haemofiltration OR hemodiafiltration OR haemodiafiltration OR "chronic kidney failure" OR "chronic renal failure" OR predialysis OR pre-dialysis OR "chronic renal" OR "chronic kidney" OR "Renal Disease" OR "Renal Diseases") AND ("motivational interviewing" OR "motivational intervention" OR counseling OR "behavioral intervention" OR "behavioural intervention" OR "educational intervention" OR "cognitive intervention" OR "psycho-social intervention" OR "psychosocial intervention" OR psychotherapy OR psychological OR educational OR psycho-educational OR psychoeducational)) AND ("randomised controlled trial" OR "controlled clinical trial" OR randomized OR placebo OR randomly OR trial OR groups)

**Cochrane Library**

(("Kidney Disease" OR "Kidney Diseases" OR "Renal Insufficiency" OR "Renal Insufficiencies" OR "Renal Replacement Therapy" OR "Renal Replacement Therapies" OR hemodialysis OR haemodialysis OR hemofiltration OR haemofiltration OR hemodiafiltration OR haemodiafiltration OR "chronic kidney failure" OR "chronic renal failure" OR predialysis OR pre-dialysis OR "chronic renal" OR "chronic kidney" OR "Renal Disease" OR "Renal Diseases") AND ("motivational interviewing" OR "motivational intervention" OR counseling OR "behavioral intervention" OR "behavioural intervention" OR "educational intervention" OR "cognitive intervention" OR "psycho-social intervention" OR "psychosocial intervention" OR psychotherapy OR psychological OR educational OR psycho-educational OR psychoeducational)) AND ("randomised controlled trial" OR "controlled clinical trial" OR randomized OR placebo OR randomly OR trial OR groups)

**Scopus**

(("Kidney Disease" OR "Kidney Diseases" OR "Renal Insufficiency" OR "Renal Insufficiencies" OR "Renal Replacement Therapy" OR "Renal Replacement Therapies" OR "hemodialysis" OR "haemodialysis" OR "hemofiltration" OR "haemofiltration" OR "hemodiafiltration" OR "haemodiafiltration" OR "chronic kidney failure" OR "chronic renal failure" OR "predialysis" OR "pre-dialysis" OR "chronic renal" OR "chronic kidney" OR "Renal Disease" OR "Renal Diseases") AND ("motivational interviewing" OR "motivational intervention" OR "counseling" OR "behavioral intervention" OR "behavioural intervention" OR "educational intervention" OR "cognitive intervention" OR "psycho-social intervention" OR "psychosocial intervention" OR "psychotherapy" OR "psychological" OR "educational" OR "psycho-educational" OR "psychoeducational")) AND ("randomised controlled trial" OR "controlled clinical trial" OR "randomized" OR "placebo" OR "randomly" OR "trial" OR "groups")

**PsycInfo**

((("Kidney Disease" OR "Kidney Diseases" OR "Renal Insufficiency" OR "Renal Insufficiencies" OR "Renal Replacement Therapy" OR "Renal Replacement Therapies" OR hemodialysis OR haemodialysis OR hemofiltration OR haemofiltration OR hemodiafiltration OR haemodiafiltration OR "chronic kidney failure" OR "chronic renal failure" OR predialysis OR pre-dialysis OR "chronic renal" OR "chronic kidney" OR "Renal Disease" OR "Renal Diseases") AND ("motivational interviewing" OR "motivational intervention" OR counseling OR "behavioral intervention" OR "behavioural intervention" OR "educational intervention" OR "cognitive intervention" OR "psycho-social intervention" OR "psychosocial intervention" OR psychotherapy OR psychological OR educational OR psycho-educational OR psychoeducational)) AND ("randomised controlled trial" OR "controlled clinical trial" OR randomized OR placebo OR randomly OR trial OR groups)

**Supplemental File 2**

a) RCTs Risk of Bias assessment.

b) Quasi-RCTs Risk of Bias Assessment.

**Supplemental File 3**

Sensitivity analysis.

**Supplemental File 4**

Funnel Plot.
